# The effect of a learning curve on outcomes of vestibular schwannoma surgery: A systematic review

**DOI:** 10.1007/s10143-026-04256-3

**Published:** 2026-04-09

**Authors:** Jacob M.C. Deeb, Hamish J.M. Dowie, Christopher Ovenden, Mendel Castle-Kirszbaum, Sharad Chawla, Michael Schultz, Stephen Santoreneos, Nick Vrodos, Nicholas G. Candy

**Affiliations:** 1 Adelaide Medical School, Faculty of Health and Medical Sciences, University of Adelaide, Adelaide, South Australia Australia; 2 Department of Surgery, University of Adelaide, Adelaide, South Australia, Australia; 3 Royal Adelaide Hospital, Adelaide, South Australia Australia; 4 Department of Neurosurgery, Monash Health, Melbourne, Australia; 5 Department of Surgery, Monash University, Melbourne, Australia; 6 Department of Otolaryngology, Southern Adelaide Local Health Network, Adelaide, South Australia Australia; 7 Department of Neurosurgery, Flinders Medical Centre, Adelaide, South Australia Australia

**Keywords:** Vestibular schwannoma, Neurosurgery, Translabyrinthine, Retrosigmoid, Middle Fossa, Learning curve, Facial nerve

## Abstract

Vestibular schwannoma (VS) surgery can be complex, with the risk of incurring significant neurological deficit or other complication. Our systematic review aimed to investigate the presence of a ‘learning curve’ for various outcomes in VS surgery. The review was conducted in accordance with PRISMA guidelines, with the Medline, Scopus, Embase, Cochrane library and Web of Science databases searched on the 30/4/25 for articles investigating the learning curve in VS surgery. Information extracted included age, gender, surgical approach, surgery duration, extent of resection, facial nerve (FN) outcome, hearing preservation (HP), complications, and further treatment. The time or patient number intervals that the learning curve was assessed at was recorded as well. The Newcastle-Ottawa Scale was utilised to determine risk of bias. Twelve studies were identified, reporting on 4059 VS cases. FN function was reported in eleven studies, all of which suggested improved FN outcomes with increased surgical experience. Hearing preservation was demonstrated to be statistically significant in four studies. Studies sporadically reported on surgical duration, resection and complication rates, with no clear learning curve identified for any of these outcomes. Our review identified the presence of a learning curve with regards to FN outcome in VS surgery with a plateau in initial surgical learning curve being obtained in the later cases of an early career surgeon’s experience. A learning curve was also found for HP in cases where this was an operative goal. Evidence demonstrating a learning curve for other outcomes is limited.

## Introduction

Vestibular schwannomas (VS) are benign neoplasms arising from the vestibular portion of the vestibulocochlear nerve [1]. Management options include surveillance, radiation therapy or microsurgical resection. Resection is generally recommended in the setting of large tumours with brainstem compression [2]. The tumour’s proximity to the facial nerve (FN) increases with risk of injury during surgery. Therefore, preservation of FN function is a key operative goal. In addition, extent of resection (EOR) is an important outcome, with the typical operative goal being gross total resection (GTR) or near total resection (NTR) [3]. In certain cases (e.g. approaches other than translabyrinthine or with serviceable hearing preoperatively) hearing preservation (HP) can also be a goal of surgery.

Originally described in aeronautical engineering, a learning curve refers to an increase in production efficiency with reduced costs as a workforce’s skill and experience increased [4]. This has been extrapolated to surgery and applies to improved patient outcomes and complication avoidance as a surgeon’s experience increases over time [5]. Originally thought to represent a sigmoid curve with three phases; an early phase I of slow skill acquisition, followed by a phase II of rapidly increasing performance and competence, transitioning to phase III of a plateau with skill mastery [6]. Contemporary research suggests that for complex surgical procedures, like skull base surgery, this phase III is dynamic and characterised by a cycle of skill mastery leading to a surgeon attempting more challenging cases, resulting in underperformance, which drives further skill refinement improving performance, which results in attempting more complex cases [7].

Candy et al. demonstrated that for endoscopic pituitary surgery the rates of GTR significantly improved with surgeon experience, and endocrine outcomes demonstrated a trend to improvement over time [8]. It is logical to assume that a similar learning curve for resection of VS exists. Individual case series have demonstrated this relationship [8–10]. However, due to the heterogeneity in the outcome reporting of these studies it is unclear if there is a consistent learning curve in VS surgery. This systematic review aims to comprehensively identify data on VS surgery and to determine the nature of the surgical learning curve and which parameters are most appropriate for accurately measuring and mapping this curve.

## Methods

### Database search

An electronic search through the databases Medline, Scopus, Embase, Cochrane Library and Web of Science was performed. The search was guided according to the preferred reporting items for systematic reviews and meta-analyses (PRISMA) 2020 [11]. The search date domain was from the inception of material until the 30th of April 2025. The following search terms were employed to identify articles reporting on the association between surgeon experience and the outcome for VS surgery: (learning curve OR Experience OR Learning) AND (Neuroma, Acoustic/surgery OR Vestibular Schwannoma OR Acoustic Neuroma OR Acoustic Schwannoma OR Acoustic Tumo* OR Acoustic Neurilemmoma* OR Neurinoma of the Acoustic Nerve* OR Ear Schwannoma* OR Acoustic nerve tumo*). Synonyms identified through the MeSH database were also included. Additional screening of relevant article’s reference list was also performed. Only English language publications were considered in this review.

### Inclusion criteria

Studies that met the criteria for review (1) recorded data on any surgical approach; (2) at minimum assessed FN function or hearing preoperatively and (3) assessed FN function or hearing post operatively. Exclusion criteria included; (1) no follow up data on the FN function or hearing loss (2) no assessment of the learning curve for FN or hearing loss outcomes.

### Data extraction

All data was reviewed independently by 2 authors (JD & HD). Discrepancies were evaluated by third author (CO). The following data was collected and correlated: Patient number, patient age, gender, surgical approach, surgery duration, FN function immediately postoperatively with House-Brackmann (HB) grading, FN function at six months or latest follow up with HB grading, HP, complication, necessity for further treatment, time interval of assessed groups, number of intervals of assessed groups, variables showing evidence of learning curve and extent of tumour resection.

### Quality assessment

Risk of bias was assessed using the modified Newcastle-Ottawa Scale for assessing quality of nonrandomised studies [12] (**see** Table 1). This tool assessed the relevant exposure of the cohort, the comparability of studies on the basis of design and the reliability of pertinent outcomes. JD and HD, both evaluated risk of bias. Discrepancies were resolved and amended amongst JD, HD and CO.

**Table 1:** Newcastle-Ottawa Scale for Assessing Quality of Nonrandomised Studies

Quality Category		Questions	Score (*)
			Yes/ No
Selection			
1	Representativeness of the exposed cohort	a) truly representative of the average _______________ (describe) in the community b) somewhat representative of the average ______________ in the community c) selected group of users e.g. nurses, volunteers d) no description of the derivation of the cohort	* *
2	Selection of the non-exposed cohort	a) drawn from the same community as the exposed cohort b) drawn from a different source c) no description of the derivation of the non-exposed cohort	*
3	Ascertainment of exposure	a) secure record (e.g. surgical records) b) structured interview c) written self-report d) no description	* *
4	Demonstration that outcome of interest was not present at start of study	a) yes b) no	*
Comparability			
1	Comparability of cohorts on the basis of the design or analysis	a) study controls for _____________ (select the most important factor) b) study controls for any additional factor (This criteria could be modified to indicate specific control for a second important factor.)	* *
Outcome			
1	Assessment of outcome	a) independent blind assessment b) record linkage c) self report d) no description	* *
2	Was follow-up long enough for outcomes to occur	a) yes (select an adequate follow up period for outcome of interest) b) no	*
3	Adequacy of follow up of cohorts	a) complete follow up - all subjects accounted for b) subjects lost to follow up unlikely to introduce bias - small number lost - > ____ % (select an adequate %) follow up, or description provided of those lost) c) follow up rate < ____% (select an adequate %) and no description of those lost d) no statement	* *

## Results

The literature search yielded an initial 2575 articles, searching through Medline, Scopus, Embase, Web of Science and Cochrane Library databases (**see Table ****2**). 2558 were initially excluded either as duplicates or because their title and abstract content did not meet predefined eligibility criteria. The most common reason for exclusion was no assessment of a learning curve. Seventeen articles proceeded to full text review [9, 10, 13–26]. Five full text articles were excluded; two because they were published in languages other than English [18, 27], and three because they failed to evaluate or discuss the surgical learning curve [21–23].

**Table 2:**
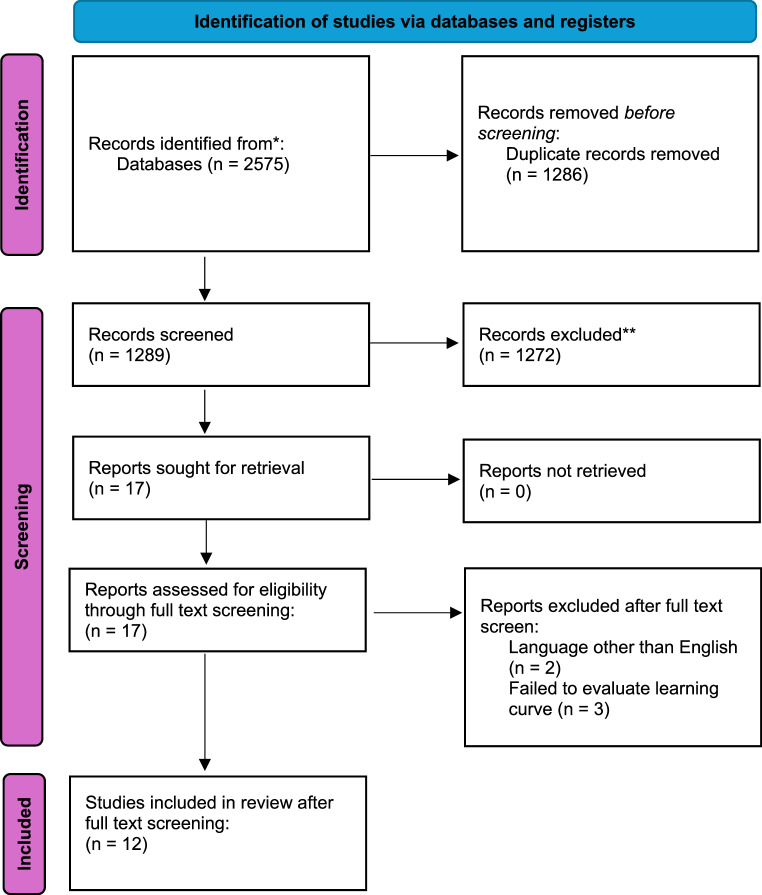
PRISMA flow diagram

### Study characteristics

**Welling et al.** [15], reported on a 160 patient cohort that underwent surgery at The Ohio State University between 1989 and 1997. The patients were sequentially allocated to eight groups of twenty patients each. FN function was assessed at more than twelve months follow up. Outcomes reported include HP, FN function, degree of tumour resection, cerebrospinal fluid (CSF) leakage (reported as rhinorrhoea), meningitis, stroke, death and recurrence or requirement for retreatment.

**Moffat et al.** [14], reported on 300 cases occurring at Addenbrooke’s Hospital from 1981 to 1995. The patients were arranged into chronological order then every 50 sequential patients were allocated into a group, forming six groups. Variables reported include FN function and EOR.

**Zhang et al.** [13], reported on 1006 patients operated on at Pitié-Salpêtrière Hospital from 1990 to 2006. The patients were grouped based on time period as early (1990–1996), intermediate (1997–2001) and late (2002–2006). Reported variables include FN function, HP, EOR, CSF leakage, mortality, septic meningitis, postoperative cerebellopontine angle haematoma, pulmonary embolism, requirement for re-treatment, subcutaneous abdominal haematoma, lower cranial nerve palsy, conservative treatment, revision, phlebitis, cerebellar oedema, lateral sinus thrombosis, subarachnoid haemorrhage.

**Heiferman et al.** [9], reported on 860 patients operated on at Loyola University Stritch School of Medicine, from 1988 to 2018. Patients were divided into three groups based on chronological order, of 400 patients (1988 to 2004), 204 (2005–2009) and 256 (2010–2018). Outcomes reported on include CSF leakage, FN function and EOR.

**Wiet et al.** [17], reported on 484 patients operated on at Evanston Hospital, from 1983 to 1999. Patients were divided into 2 groups based on chronological order as fourteen cases for patients between 1983 and 1992 and fifteen cases for patients between 1994 and 1999. Variables reported on include HP, FN function and EOR.

**Buchman et al.** [16], reported on 96 patients who underwent VS surgery at Pittsburgh Ear Associates and Allegheny General Hospital, from November 1987 to June 1995. Patients were chronologically numbered from one to 96 and divided into four groups with twenty sequential patients in each with sixteen in the fifth group. Variables reported on include CSF leakage, bacterial meningitis, stroke, HP, repeat surgery, operative time, FN function and EOR.

**Elsmore et al.** [10], reported on 127 patients who underwent VS surgery at Charing Cross Hospital, from 1972 to 1995. Patients were allocated into three groups based on chronological order, for every seven years: 23 (1972–1979), 31 (1980–1987) and 73 (1988–1995). Variables reported on include, FN function, HP, mortality, CSF leakage and EOR.

**Hudelist et al.** [28], reported on 100 patients from Pitié-Salpêtrière Hospital. This study was part of a series of three studies with homogenous data. As a result, we included the two prior studies [29, 30] allowing for examination of the learning curve from 2009 to 2022. Patients were grouped by chronological order: 229 (2009–2011), 25 (2014), and 100 (2022–2023) FN function was reported at greater than six months follow up. Reported variable include EOR, FN function and requirement for further surgery.

**Kanzaki et al.** [20], reported on 127 patients operated on at Keio University Hospital, from 1976 to 2000. Patients were ordered chronologically in groups for the periods 1976–1988, 1989–1994, 1995–1999. Outcomes reported include HP, incidence of intracanalicular tumours and tumour size.

**Wang et al.** [24], reported on 153 patients, operated at Westmead hospital, from 1999 to 2011. Patients were ordered by case sequentially to form a cumulative distribution. This was then mapped as a learning curve, cumulative summation model. Outcomes reported include FN outcome, NF2, complete resection, CSF leak, transection of the facial nerve, meningitis, stroke, seizure, postoperative wound infection.

**Foroughi et al.** [26], reported on 102 patients, operated at James Cook University Hospital, Middlesborough UK, from 1986 to 2000. Patients were ordered sequentially to form a cumulative summation model. Outcomes reported include age and sex of patients, tumour size, pre and post-op FN function, macroscopic preservation of facial nerve, length of operation, use of FNM, use of the KTP-532 (potassium titanyl-phosphate) laser and complications.

**Schackert et al.** [25], reported on 544 patients, operated on at the Department of Neurosurgery, Technische Universität, Dresden, from 1991 to 2019. Patients were ordered sequentially and group every decade, for the periods 1991–1999, 2000–2009, 2010–2019. Data collected includes: EOR, operative duration, tumour size, positioning of the patient, FN function, HP, CSF leakage, postoperative haemorrhage, pulmonary embolism and development of compartment syndrome.

### Patient demographics

Patient demographics are reported inconsistently across studies. Reported variables include age, gender, surgical approach, time interval of assessed groups, and number interval of assessed groups. These are reported in [Table 3].

**Table 3:** Study Characteristics

Study (Author, Year of Publication)	Patients Included in Study	Patient Age & Sex	Surgical Approach (TL: Translabyrinthine) (MF: Middle Fossa) (RS: Retrosigmoid)	Time Interval of Assessed Groups	Number Interval of Assessed Groups
*D.B. Welling et al, 1999*	160	Mean age: 52 Male: 79F emale: 81	TL: 61.9% Suboccipital: 17% MF: 16.2% TL/Suboccipital: 5%	Assessed chronologically in groups of 20 (8 total groups) between 1989 and 1997	8 groups of 20 in sequential order
*D.A Moffat et al, 1996*	300	Mean age:51.75 Sex not reported	TL: 80% RS: 20%	Assessed chronologically in groups of 50 between 1981 and 1995	6 groups of 50 in sequential order
*Z. Zhang et al, 2016*	1006	Mean age: 55.6 Male: 472 Female: 534	TL: 64% Transotic: 10% RS: 18% MF: 8%	Group 1:1990-1996 Group 2: 1997-2001 Group 3: 2002-2006	Group 1:268 patients Group 2: 299 patients Group 3: 439 patients
*D.M Heiferman et al, 2023*	860	Mean age: 50 Male: 426 Female: 434	RS & TL for tumours less than 1.5cm, RS &TL combined for tumours larger than 4cm, MF used sparingly	Group 1:1988-2004 Group 2: 2005-2009 Group 3: 2010-2018	Group 1: 400 patients Group 2: 204 patients Group 3: 256 patients
*R.J. Wiet et al, 2000*	484	Mean age: 50.4 Male: 278 Female: 206	TL: 79% MF: 7% RS/Suboccipital: 14%	Group 1: 1983 – 1992 Group 2: 1994 - 1999	Group 1: 14 patients Group 2: 15 patients
*C.A. Buchman, et al, 1996*	96	Mean age: 50 Male: 55 Female: 41	TL: 64%RS: 17% MF: 13% Combined: 6%	Assessed chronologically in groups of 20 (5 total groups) between 1987 and 1995	5 Groups of 20 in sequential order
*A.J. Elsmore, N.D Mendoza, 2002*	127	Mean age: 49.6 Male: 55 Female: 72	RS: 100%	Group1: 1972 – 1979 Group 2: 1980 – 1987 Group 3: 1988 – 1995	Group 1: 23 patients Group 2: 31 patients Group 3: 73 patients
*B. Hudelist et al, 2025*	100	Not reported	RS: 85% TL: 15%	Group1: 2009-2011 Group 2: 2014 Group 3: 2022-2023	Group1: 229 patients Group 2: 25 patients Group 3: 100 patients
*J. Kanzaki et al, 2001*	127	Mean age: 46 Sex not reported	MF/Extended MF: 100%	Group1: 1976 – 1988 Group 2: 1989 – 1994 Group 3: 1995 - 1999	Group1: 14 patients Group 2: 62 patients Group 3: 51 patients
*A.Y. Wang et al., 2013*	153	Mean age: not reported Male: 69 Female: 84	TL: 93% RS: 4%MF: 3%	Cumulative distribution (1999-2011)	
*M. Foroughi et al., 2010*	102	Not reported	RS: 100%	Cumulative distribution (1986-2000)	
*G. Schackert et al., 2020*	544	Mean age: 57 Male: 245 Female: 299	RS: 100%	Group1: 1991-1999 Group 2: 2000-2009 Group 3: 2010-2019	Group1: 103 patients Group 2: 210 patients Group 3: 231 patients

### Outcome findings

Outcome findings are reported in [Table 4].

**Table 4:** Outcome Findings

Study (Author, Year of Publi.)	Surgery Duration	Facial Nerve Function:	Hearing Preservation	Complications	Resection quality	Further Treatment
*D.B. Welling et al, 1999*	Reduced from 13.5 hour mean to 5.9hour mean across study duration.	Significiant improvement for HB1 from first group to ensuing groups (35% vs 74%). No statistical significance for HB1/2. Overall effect of group number on FN function (p=0.034).	Group 1: 50% excellent, 50% poor Group 3:57% excellent, 14% poor Group 5:57% excellent, 43% poor Group 7: 50% excellent, 50% poor Group 8: 20% excellent, 80% poor. No statistical significance reported.	CSF leak in 40% of patients in group 1 compared with 5% of patients in group 8. Statistical significance not reported.	GTR: 97. 5% NTR: 2.5%. No statistically significant change.	4 recurrence noted. 2 treated with MF approach, 1 treated with TL approach, 1 conservative management.
*D.A Moffat et al, 1996*	Not reported	Statistically significant improvement in HB1 between first and second group (26% vs 34%, p=0.02). Statistically significant improvement between the fifth and sixth group (32% vs 56%, p=0.01).	Not Reported	No statistically significant improvement	GTR: 100%	1 recurrence noted
*Z. Zhang et al, 2016*	Not Reported	Statistically significant improvement in HB 1/2 FN function between first and last period (78.4% vs 87.6%, p=0.02).	Hearing preservation rate: 50.9% (Group 1/2) vs 69.0% (Group 3), p=0.028. Serviceable hearing (Class A + B, AAO-HNS) preservation rate: 21.8% (Group 1/2) vs 42.2% (Group 3), p=0.01.	Mortality rate improved from 0.4% in group 1 to 0.2% in group 3.CSF leakage rate improved from 11.6% to 7.1% in group 3. Subcutaneous abdominal hematoma 3.4% in group 1 to 1.1% in group 3.	GTR: 99.4% NTR: 0.6%. No statistically significant change.	12 recurrence noted, 19 revision surgeries.
*D.M Heiferman et al, 2023*	Not Reported	Statistically significant improvement in HB1/2 between first period, second period and third period (78.3% vs 92.8% vs 87.9%).Overal improvement of FN function with time (p<0.001).	Not Reported	No statistically significant improvement	GTR: 700 NTR: 55 STR: 82. GTR decreased from 94.4% to 88.7% to 63.7% across groups. STR increased from 0.3% to 7.4% to 25.8% across groups. Statistical significance was not reported.	N/A
*R.J. Wiet et al, 2000*	Not Reported	Statistically significant improvement of FN preservation over time (p<0.05).	Class A hearing preserved in 36% of cases in the first 10 years, compared with 66% of cases in the last 5 years. Significant improvement of HP over time (p<0.05)	No statistically significant improvement	All STR excluded.	N/A
*C.A. Buchman, et al, 1996*	Average operating time for tumours less than 1cm: 401 minutes Average operating time for tumours larger than 2.5cm: 622 minutes.	HB grade 1/2 observed in 50% of patients in group 1, compared with 100% of patients in group 5. FN function improvement with time was statistically significant (p=0.0003).	“hearing results have improved over time.”	No statistically significant improvement	GTR: 77% NTR: 19% STR: 6%. No statistically significant change.	Recurrence noted in 6% of cases
*A.J. Elsmore, N.D Mendoza, 2002*	Not Reported	HB grade 1/2 observed in 17.4% of patients in group 1 vs 56% of patients in group 2. Statistical significance for FN function improvement over time evident only for medium size tumours (p=0.05).	No statistically significant improvement	No statistically significant improvement	Not Reported	N/A
*B. Hudelist et al, 2025*	Average operating time: 233 minutes	HB grade 1/2 observed in 75% of patients with stage III/IV tumour in group 1 and in 98% of patients with stage III/IV tumour in group 3. Statistical significance compared with the FN function out come in group 3 was p<0.001 in group 1 and p=0.0145 in group 2.	No statistically significant improvement	No statistically significant improvement	GTR: 17 NTR: 22 STR: 52 PR: 9. Intentional change toward NTR/STR, no statistical significance reported.	Recurrence noted in 17% of cases
*J. Kanzaki et al, 2001*	Not Reported	Not reported	Pre operative Class A hearing preserved in 33% in first group compared with 67% in last group. Signficant improvement of HP over time (p<0.05).	No statistically significant improvement	Not Reported	N/A
*A.Y. Wang et al., 2013*	Not reported	Reached statistically significant level of competence at 56^th^ procedure (p<0.05).	Not reported	No statistically significant improvement	No statistically significant change	Not reported
*M. Foroughi et al., 2010*	Not reported	Reached statistically significant level of competence at 27^th^ procedure (p<0.05).	Not reported	No statistically significant improvement	Not reported	Not reported
*G. Schackert et al., 2020*	Statistically significant decrease from 1^st^ to last group (P<0.001) for T3/T4 tumours.	Statistically significant improvement when comparing the 1^st^ to the 3^rd^ group (P<0.001).	Statistically significant improvement between 1^st^ to 3^rd^ group (p=0.030), more significant improvement for small T1/T2 tumours (p=0.019).	Statistically significant reduction in blood loss for T3/T4 tumours comparing 1^st^ to 3^rd^ group (p<0.001). Statistically significant reduction in wound infection comparing 1^st^ to 3^rd^ group (p=0.004). Statistically significant improvement in postoperative hemorrhage in in the last decade (p-0.027).	No statistically significant change	Not reported

### Facial nerve outcome

FN function was reported in eleven articles, all of which demonstrated statistically significant improvement with experience. Reported thresholds ranged widely (14-1006 cases) and were based on heterogenous definitions and grouping strategies. Traditionally one of four methodologies are used to analyse a surgical learning curve: graphical inspection, split grouping, cumulative sum method and regression [31]. Among the papers we reviewed, the favoured reporting method was split grouping. Whereby the plateau of the learning curve was reported as the point at which there was a statistically significant difference in the FN outcome between groups or eras. Only two articles differed from this trend, favouring the cumulative sum method [24, 26]. Six articles reported the case threshold as a range (as era or group) [10, 13, 14, 17, 25, 28]. Five articles reported a specific case threshold as well as a range (as era or group) [9, 15, 16, 24, 26].

To determine the total mean case threshold, the mean of the reported specific case thresholds (119.8), and the mean of the median number within each reported threshold range was calculated (236). The total range was determined as the mean range in each series that reported case threshold as a group or era only (238). This method was adapted from Shultz et al. who similarly reviewed heterogeneously reported surgical learning curves [32]. As a result, the mean number of cases required to reach statistically significant improvement in FN outcomes was 178. However, the authors recognise that the true number may lie anywhere from fourteen to 416 cases.

### Operative duration

Operative duration was reported in three articles [15, 16, 28]. Welling et al. reported a reduction in operative time from a mean of 13.5 to 5.9 hours when comparing the first to last group. The remaining articles by Hudelist et al. and Buchman et al. commented on overall improvements with outcomes trend varying, due to heterogeneity of tumour characteristics (e.g. size).

### Extent of resection

EOR was reported in seven articles. Three studies [14, 15, 24], pursued a goal of GTR wherever possible. No statistically significant improvement was demonstrated. Three studies pursued or altered their surgical goal from GTR, to STR or NTR, prioritising FN preservation as time progressed. This was reflected in lowered rates of GTR overall [9, 16, 28]. Heiferman et al. initially focused on GTR in their series but later shifted to NTR with FN preservation. GTR decreased from 94.4% to 88.7% in the first two groups, to 63.7% in the last. A similar shift in philosophy was seen in the papers by Buchman et al. and Hudelist et al. One study [17] excluded STR; and did not differentiate between NTR and GTR, making it difficult to assess EOR association with outcomes in this study.

### Hearing preservation

Outcomes for HP were reported in six studies. Five studies depicted the improvement in HP at each patient interval [13, 15, 17, 20, 25]. Of these studies Zhang et al., Wiet et al. and Kanzaki et al. utilised the American Academy of Otolaryngology-Head and Neck Surgery (AAO-HNS) hearing class to report on HP. Welling et al. [15] categorised hearing outcomes as excellent (pure tone decreased by less than 10dB and discrimination by less than 20%), serviceable, and poor (both being less than 50dB decrease in pure tone, and greater than 50% decrease in discrimination). Schackert et al. reported hearing outcomes using the Gardner- Robertson scale. Four of these studies [13, 17, 20, 25] had reported statistically significant improvement when comparing group one or two with the last group (0-299 to 51–567 cases). This suggests a more prolonged phase I, but still demonstrated a sigmoid like trend. One study [16], made general comment on HP improvement with experience. The remaining six studies did not report HP outcomes [9, 10, 14, 24, 26, 28].

### Complications

Complications were reported in seven articles [9, 10, 13, 15, 16, 24, 25]. CSF leakage decreased incidence across two articles [13, 15] and showed no statistically significant change across the remaining three [9, 10, 24]. Mortality was reported across three articles [10, 13, 15] and showed no statistically significant change in mortality rate with experience. Schackert et al. [25] denoted statistically significant improvement in reduced blood loss for T3/T4 sized tumours over time. This is in addition to wound infection and post-operative haemorrhage improving over time. Other complications were reported with no statistically significant evidence of a learning curve.

### Additional treatment

Requirement for additional treatment was reported in five studies [13–16, 28]. No consistent evidence was found for a learning curve with respect to this outcome.

### Surgical approach

Wang et al. [24] found that translabyrinthine approach was statistically significant for association with improvements in FN outcome at one year postoperative follow up. Of the 153 patients in their article, 93% of patients underwent a translabyrinthine approach, with 4% undergoing RS and 3% undergoing MF.

### Study quality

Overall study quality was determined to be good quality [Table 5] across all articles, in accordance with the Agency on Healthcare Research and Quality standards [33]. Common features of quality deficit include: the assessment of outcome being self-reported. This is in addition to a lack of description relating to the selection of the non-exposed cohort, however, is not pertinent to this case series.

**Table 5:** Risk of Bias Assessment Results

Authors (year)	Study Type	Selection				Comparability	Outcome			Final Score
		Representativeness of the exposed cohort	Selection of the non-exposed cohort	Ascertainment of exposure	Demonstration that outcome of interest was not present at start of study	Comparability of cohorts on the basis of the design or analysis	Assessment of outcome	Was follow-up long enough for outcomes to occur	Adequacy of follow up on cohorts	
*D.B. Welling et al.*,* 1999*	Retrospective cohort	*		*	*			*	*	*****
*D.A Moffat et al.*,* 1996*	Retrospective cohort	*		*	*			*	*	*****
*Z. Zhang et al.*,* 2016*	Retrospective cohort	*		*	*			*	*	*****
*D.M Heiferman et al.*,* 2023*	Retrospective cohort	*		*	*			*	*	*****
*R.J. Wiet et al.*,* 2000*	Retrospective cohort	*		*	*			*	*	*****
*C.A. Buchman*, et al.,* 1996*	Retrospective cohort	*		*	*			*	*	*****
*A.J. Elsmore*,* N.D Mendoza*,* 2002*	Retrospective cohort	*		*	*			*	*	*****
*B. Hudelist et al.*,* 2025*	Retrospective cohort	*		*	*			*	*	*****
*J. Kanzaki et al.*,* 2001*	Retrospective cohort	*		*	*			*	*	*****
*A.Y. Wang et al.*,* 2013*	Retrospective cohort	*		*	*			*	*	*****
*M. Foroughi et al.*,* 2010*	Retrospective cohort	*		*	*			*	*	*****
*G. Schackert et al.*,* 2020*	Retrospective cohort	*		*	*			*	*	*****

## Discussion

This article has examined the association between outcome metrics and the effects of increasing surgical experience on these metrics over time. This systematic review finds a correlation between greater surgical expertise and improved key operative outcomes, including FN function and HP rates. These improvements may be related to a surgeon’s learning curve as they master this complex surgical procedure.

### Facial nerve outcome

The impact of a learning curve on FN outcomes is complex as it may be confounded by changes in surgical philosophy of how aggressively surgeons pursued GTR. Traditional surgical philosophy has emphasized GTR, potentially at the expense of suboptimal FN outcomes [34]. However, ongoing discussion regarding the superiority of partial tumour removal followed by radiotherapy, over complete removal [35] has in some centres shifted toward FN preservation over GTR. This philosophical change may have impacted the results of some included articles [9, 13, 25, 28], but the magnitude of the effect is unclear. Multiple articles [10, 14–17, 24, 26] have shown a learning curve for FN outcomes without an explicit change in operative goals. Despite this, it is still possible a change in operative goals within studies was associated with FN outcome, thereby confounding effects to define the learning curve for this outcome. Though not possible with our data set, it would be interesting to assess how surgical aim influences the learning curve: does aiming for a NTR result in an altered slope of the learning curve when compared to a GTR?

The authors recognise that the heterogeneity in reported case thresholds and the differing methods of reporting surgical learning curve result in great degree of uncertainty. Without homogenous reporting methodology, and even with sophisticated methods of calculation, the pooled threshold may not be clinically relevant due to its large range. Further to this, in some cases, where a specific case threshold was reported, this was not based on a specific learning curve analysis methodology. But rather, these were reported based on arbitrary measures determined by the studies’ authors.

### Surgical duration

Three articles [15, 16, 28], demonstrated an improvement in surgical duration, with surgeon experience. In the case of Welling [15] and Buchman [16] the translabyrinthine was the predominant approach (61.9% and 64% respectively), hence the improvement in surgical duration may be attributable to the improvement in translabyrinthine approach. In contrast, the improvements in surgical duration in the predominant retrosigmoid approach, performed by Hudelist et al. [28] were more likely attributable to improvements in microsurgical resection. Similar improvements in surgical duration with experience have been identified in other surgical procedures, such as laparoscopic nephrectomy [36].

### Hearing preservation

Four articles reported statistically significant improvements in HP outcomes with increasing surgeon experience [13, 17, 20, 25]. In these articles, HP outcomes generally reached plateau in improvement later within the case series (14–567 patients) [37]. It is well established that the probability of HP is significantly influenced by: the size of the tumour, preoperative hearing level, and type of surgical approach [38]. The most common surgical approach between the four papers was the retrosigmoid approach. Tumour size was commonly ≤ 15 mm, and the majority of patients presented with a preoperative hearing of Class A. Despite this, it must be considered that the differing percentage of patients with functional hearing before surgery differed among studies. The included articles were not case series reporting specifically on HP, but rather a case series of operated VS. As a result, it may underappreciate the degree to which surgical experience influences HP outcomes. These findings must be placed in the context of contemporary practice placing greater emphasis on HP if achievable, potentially confounding these results. This suggests that a learning curve may exist with regards to hearing preservation in those cases that it is appropriate [26].

### Learning Curve

All teams were comprised of surgeons who had completed neurosurgical training, with some completing further lateral skull base fellowship training as well as otolaryngological surgeons with subspecialty skills in lateral skull base surgery. Although many of the countries reported do have formal neurotologic training fellowships it is not possible to determine whether the standard of training is equivalent between each program. Studies did not clearly delineate which procedures solely involved neurosurgeons vs those done as a combined otolaryngology/neurosurgeon operation. The inconsistency in the members of the operative team introduces some degree of confounding to the learning curve that is difficult to quantify as included studies did not report on this data. It is possible that the neuro-otological stage vs the neurosurgical stages of VS surgery have a learning curve unalike in its morphology. This is discussed explicitly by Wang et al. [24] who demonstrated a, “learning curve [relevant] of [the] neurological team.” Delineation of each member’s individual learning curve warrants further review, and there was limited analysis of the relative personal experience of the members of the surgical team in each study.

The learning curve is impacted by many variables that are not well documented in the literature. Some factors that were not analysed in the included studies and could confound outcomes include: volume of surgical cases performed at one institution, patient demographics, tumour factors (size, lateral extent, vascularity, cystic features), whether patients in one series were operated on by the same surgeon, quality of surgical field, post operative lateral and sigmoid sinus thrombosis, and anatomical arrangements of the temporal bone and cerebellopontine angle.

Addressing this learning remains the inherited burden of young neurosurgeons and neurotologist. Roser et al. [39], highlights the beneficial steps a young surgeon might take to address this: training consisting of anatomical dissection courses, recollection of experienced surgeons’ surgeries, early hospitation after a certain level of training, engagement in clinical research relating to skull based procedures. This is in addition to developing skills such as recognition of the trigemino-cardiac reflex, preservation of the petrosal vein, endoscopic assistance for intrameatal tumour aspects, preservation of the semicircular duct and usage of vasoactive substance to preserve cochlear nerve function.

Novel research [40, 41] into the use of surgical simulation, videos and augmented reality technology are promising adjuncts that could potentially accelerate the surgical learning curve. Pöser et al. [42] highlights the potential for simulation models to accelerate a surgeon’s learning curve. This was demonstrated by their research into the benefits of a 3D tutorial for spinal lumbar herniation surgery, followed by non-cadaveric simulated practice. This study concluded a 30% improvement in microsurgical performance, as well as significant improvement in mental conceptualisation of the surgery. Similar models of training may accelerate the learning curve in VS surgery. Ledwos et al. [43] recently demonstrated the utility of artificial intelligence (AI) models in differentiating skill levels and tracking performance in subpial resection. The AI model developed metrics to assess trainee’s performance which were subsequently incorporated into personalised feedback. This resulted in an improved learning curve. Models such as these could feasibly be integrated into VS surgical training, and future research could focus on this.

If more research is to be conducted into mapping the learning curve for VS surgery, a more consistent and refined scope toward reported surgical outcomes is required. More homogenous data will allow development of more robust models of the learning curve with relation to specific surgical outcomes. The data that is utilised in future models needs to be standardised with competence clearly defined. This could subsequently potentiate the training of surgeons to overcome this learning curve.

### Limitations

This review’s primary limitations related to the heterogeneity of data, which was reported and collected. The retrospective nature and potential bias related to author choice of outcomes measured explored limits this review. This is further confounded by the shift in surgical philosophy in certain studies, potentially undermining the ability for isolated evaluation of the learning curve. Limited consistency in reporting of surgical outcomes among the three standard approaches prevents cross technique comparison. The authors acknowledge that varying approaches require different skill sets and the consideration of different anatomical obstacles. It was assumed in analysing the learning curve with respect to FN and HP that the surgical teams have selected the best available approach for these goals. A surgeon’s prior experience before the beginning of their learning-curve assessment in this study was not well documented and could bias the results. With the advent of stereotactic radiosurgery (SRS), it is also possible that the proportion of cystic tumours in surgical series may have increased given they are poor SRS candidates. Cystic tumours are known to be associated with worse FN outcomes, and given the included series did not report on this factor it may have confounded results [44]. Lastly, it must be considered that the series by Hudelist et al. and Zhang et al. reported some data from the same institution. Due to paucity in the definition of which surgeons performed each procedure, potential overlapping data may have been reported.

## Conclusion

This review confirms the existence of a learning curve in VS surgery with regards to FN function and HP. Post operative FN function is therefore an outcome that can be used to track progression of a surgeon’s learning curve. Future research with homogenous standards for outcome reporting will help to better map the learning curve in VS surgery. Notably, the inferior outcomes observed early in a surgeon’s operative career highlight the need to develop training models that accelerate progression along the learning curve.

## Data Availability

No datasets were generated or analysed during the current study.

## Declarations

### Clinical trial number

Clinical trial number: not applicable.
